# Supplementary material for: Adolescents and adults with FOXP1 syndrome show high rates of anxiety and externalizing behaviors but not psychiatric decompensation or skill loss
Source: Front Psychiatry. 2025 Feb 24;16:1526383. doi: 10.3389/fpsyt.2025.1526383 (PMC11891879; doi:10.3389/fpsyt.2025.1526383)
Supplement: SUPPLEMENTARY DATA SHEET 1 — Visual analog scale. [file DataSheet1.pdf]

Visual Analog Scale (VAS)

Please complete the survey below.

Thank you!

Please list the top three concerns you had about your child during his/her early childhood.

Childhood Concern #1 ______________________

Please rate this concern as it presented during early childhood.

0 50 100

(Place a mark on the scale above)

Please rate this same concern now, as an adolescent/adult.

0 50 100

(Place a mark on the scale above)

Childhood Concern #2 ______________________

Please rate this concern as it presented during early childhood.

0 50 100

(Place a mark on the scale above)

Please rate this same concern now, as an adolescent/adult.

0 50 100

(Place a mark on the scale above)

Childhood Concern #3 ______________________

Please rate this concern as it presented during early childhood.

0 50 100

(Place a mark on the scale above)

Please rate this same concern now, as an adolescent/adult.

0 50 100

(Place a mark on the scale above)

Please list the top three concerns you have about your child now, as an adolescent/adult.

Adolescent/Adult Concern #1 ______________________

Please rate this concern.

0 50 100

(Place a mark on the scale above)

Adolescent/Adult Concern #2 ______________________

Please rate this concern.

0 50 100

(Place a mark on the scale above)

Adolescent/Adult Concern #3

____

Please rate this concern.

0 50 100

(Place a mark on the scale above)
